# Complete Genome Sequences of 10 Yersinia pseudotuberculosis Isolates Recovered from Wild Boars in Germany

**DOI:** 10.1128/genomeA.00266-18

**Published:** 2018-05-10

**Authors:** Marie Reinhardt, Jens A. Hammerl, Stefan Hertwig

**Affiliations:** a German Federal Institute for Risk Assessment, Berlin, Germany

## Abstract

We report here the draft genome sequences of 10 Yersinia pseudotuberculosis isolates recovered from tonsils of wild boars hunted between 2015 and 2016 in Germany. Whole-genome sequencing and bioinformatic analyses were performed to assess the diversity of Y. pseudotuberculosis, which may result in human infections caused by the consumption of game meat.

## GENOME ANNOUNCEMENT

The genusYersinia comprises 18 species, of which 3 are pathogenic for humans.Y. pestis is the causative agent of plaque, while Y. enterocolitica and Y. pseudotuberculosis cause an intestinal disease called yersiniosis, for which typical symptoms are diarrhea, abdominal pain, and fever (1). Enteropathogenic Yersinia spp. are commonly ingested via contaminated food, but infections may also occur by direct contact with infected animals. Whereas the main reservoirs of Y. enterocolitica are pigs, Y. pseudotuberculosis is found predominantly in wildlife-like rodents and game (1–3). The numbers of infections caused by Y. pseudotuberculosis are much lower than those documented for Y. enterocolitica. However, some Y. pseudotuberculosis outbreaks have been reported in Finland, Norway, France, and New Zealand (4–8).

Since there is only scarce information available about the occurrence and properties of Y. pseudotuberculosis in wildlife, the prevalence of this species in tonsils of wild boars hunted in Mecklenburg-Western Pomerania (Germany) was determined. Tonsil samples of 503 wild boars, hunted between 2015 and 2016, were investigated by *wzz*-PCR and cultural detection using a cold enrichment procedure. Y. pseudotuberculosis was detected in 6.4% of the tonsils by PCR and could be isolated from 10 animals.

Here, we report the draft genome sequences and some genetic characteristics of the isolates. Genomic DNA of the isolates was prepared from bacteria grown in lysogeny broth for 24 h at 28°C using the PureLink genomic DNA minikit (Invitrogen, Carlsbad, CA, USA) according to the manufacturer's recommendations. Short-read sequencing (2 × 251 cycles) using the MiSeq V3 (600-cycle) reagent kit was used on an Illumina MiSeq benchtop sequencer (Illumina, San Diego, CA, USA). Sequencing libraries were conducted using the Illumina Nextera XT DNA sample preparation kit. Raw read data were used for *de novo* genome assembling using the PATRIC database (https://patricbrc.org) (9). SPAdes assembly calculations resulted in 10- to 20-fold sequence coverages per consensus sequence for all isolates. Genome annotation using the automated Prokaryotic Genome Annotation Pipeline (PGAP) of the NCBI-database (https://www.ncbi.nlm.nih.gov/genome/annotation_prok) revealed that the Y. pseudotuberculosis genomes exhibit only little variability in genome sizes, which range from 4.58 (M129) to 4.77 Mb (M489). Additionally, the numbers of genes, coding sequences (CDSs), RNA genes, and pseudogenes, as well as the presence of clustered regularly interspaced short palindromic repeat (CRISPR) loci, are also similar among the isolates. Data on some genetic properties of the isolates are summarized in Table 1. The gene content of the isolates is similar to that of previously described Y. pseudotuberculosis strains. Further bioinformatic analyses revealed the presence of mobile genetic elements (i.e., prophages and plasmids), which may be involved in the genome plasticity of the isolates (10–12). Further studies on the biotype, serotype, virulence gene content, and phylogenetic relationship of the isolates are necessary to assess the diversity and pathogenic potential of Y. pseudotuberculosis in wild boars in Germany.

**TABLE 1:** Some characteristics and accession numbers of the Y. pseudotuberculosis isolates reported here

Characteristic	Result(s) for isolate:									
	M66 (LFB2015W0M66/I75)	M68 (LFB2015W0M68/I79)	M69 (LFB2015M0M69/I85)	M89	M90 (LFB2015M1M90/I8)	M102 (LFB2015M0M102/I1)	M126 (LFB2016M0M126/I13)	M129 (LFB2016W1M129/I2)	M207	M489
Genome size (bp)	4,738,757	4,765,035	4,706,236	4,670,266	4,569,157	4,732,574	4,628,486	4,580,728	4,656,234	4,770,340
No. of contigs	237	158	203	226	265	112	233	149	227	275
No. of genes (total)	4,375	4,373	4,314	4,355	4,181	4,309	4,251	4,143	4,390	4,478
No. of genes (coding)	4,111	4,145	4,071	4,123	4,101	4,105	4,148	4,047	4,164	4,128
No. of CDSs (total)	4,283	4,279	4,229	4,262	3,927	4,213	3,940	3,923	4,294	4,380
No. of CDSs (coding)	4,111	4,145	4,071	4,123	3,927	4,105	3,940	3,923	4,164	4,128
No. of RNA genes (total)	92	94	85	93	80	96	103	96	96	98
No. of rRNAs (5S, 16S, 23S)	7, 2, 1	8, 3, 1	5, 3, 1	4, 3, 2	3, 2, 2	7, 3, 1	5, 3, 4	7, 3, 1	4, 4, 3	5, 4, 1
No. of tRNAs	72	72	69	73	64	74	77	73	75	74
No. of noncoding RNAs	10	10	7	11	9	11	14	12	10	14
No. of pseudogenes (total)	172	134	158	139	174	108	208	124	130	252
No. of pseudogenes with ambiguous residues	0	0	0	0	0	0	0	0	0	0
No. of frameshifted pseudogenes	30	31	29	82	36	30	33	33	71	127
No. of incomplete pseudogenes	138	96	117	53	136	74	171	88	55	122
No. of pseudogenes with internal stops	9	13	18	32	6	8	9	8	26	59
No. of pseudogenes with multiple problems	5	6	6	25	4	4	5	5	20	50
No. of CRISPR arrays	2	3	2	3	2	2	2	2	2	4
No. of predicted prophages	10	8	10	5	7	4	6	5	8	7
No. of predicted plasmids	1	1	1	1	1	1	ND^a^	ND	ND	1
Database accession no.										
GenBank	MNKQ00000000	MNKR00000000	MAKS00000000	NCLA00000000	MAKT00000000	MNKT00000000	MAKU00000000	MAKV00000000	NCKY00000000	NCLF00000000
BioProject	PRJNA326833	PRJNA326834	PRJNA326835	PRJNA381964	PRJNA326837	PRJNA326838	PRJNA326839	PRJNA326840	PRJNA381953	PRJNA381962
BioSample	SAMN05292970	SAMN05292970	SAMN05292972	SAMN06697619	SAMN05292974	SAMN05292975	SAMN05292976	SAMN05292977	SAMN06697573	SAMN06697618

a ND, not detected.

### Accession number(s).

The whole-genome sequences of the isolates reported here have been deposited in GenBank under the accession numbers given in Table 1.

## References

[1] Wren BW 2003 The yersiniae—a model genus to study the rapid evolution of bacterial pathogens. Nat Rev Microbiol 1:55–64. doi:10.1038/nrmicro730. 15040180

[2] Galindo CL, Rosenzweig JA, Kirtley ML, Chopra AK 2011 Pathogenesis of *Y. enterocolitica* and *Y. pseudotuberculosis* in human yersiniosis. J Pathog 2011:182051. doi:10.4061/2011/182051. 22567322 PMC3335670

[3] Laukkanen-Ninios R, Fredriksson-Ahomaa M, Maijala R, Korkeala H 2014 High prevalence of pathogenic *Yersinia enterocolitica* in pig cheeks. Food Microbiol 43:50–52. doi:10.1016/j.fm.2014.04.016. 24929882

[4] Deacon AG, Hay A, Duncan J 2003 Septicemia due to *Yersinia pseudotuberculosis*—a case report. Clin Microbiol Infect 9:1118–1119. doi:10.1046/j.1469-0691.2003.00746.x. 14616729

[5] Jalava K, Hallanvuo S, Nakari U-M, Ruutu P, Kela E, Heinäsmäki T, Siitonen A, Nuorti JP 2004 Multiple outbreaks of *Yersinia pseudotuberculosis* infections in Finland. J Clin Microbiol 42:2789–2791. doi:10.1128/JCM.42.6.2789-2791.2004. 15184472 PMC427876

[6] Parn T, Hallanvuo S, Salmenlinna S, Pihlajasaari A, Heikkinen S, Telkki-Nykanen H, Hakkinen M, Ollgren J, Huusko S, Rimhanen-Finne R 2015 Outbreak of *Yersinia pseudotuberculosis* O:1 infection associated with raw milk consumption, Finland, spring 2014. Euro Surveill 20. doi:10.2807/1560-7917.ES.2015.20.40.30033. 26537540

[7] Vincent P, Leclercq A, Martin L, Yersinia Surveillance N, Duez JM, Simonet M, Carniel E 2008 Sudden onset of pseudotuberculosis in humans, France, 2004–05. Emerg Infect Dis 14:1119–1122. doi:10.3201/eid1407.071339. 18598636 PMC2600338

[8] Vasala M, Hallanvuo S, Ruuska P, Suokas R, Siitonen A, Hakala M 2014 High frequency of reactive arthritis in adults after *Yersinia pseudotuberculosis* O:1 outbreak caused by contaminated grated carrots. Ann Rheum Dis 73:1793–1796. doi:10.1136/annrheumdis-2013-203431. 23852698

[9] Wattam AR, Brettin T, Davis JJ, Gerdes S, Kenyon R, Machi D, Mao C, Olson R, Overbeek R, Pusch GD, Shukla MP, Stevens R, Vonstein V, Warren A, Xia F, Yoo H 2018 Assembly, annotation, and comparative genomics in PATRIC, the all bacterial bioinformatics resource center. Methods Mol Biol 1704:79–101. doi:10.1007/978-1-4939-7463-4_4. 29277864

[10] Hammerl JA, Freytag B, Lanka E, Appel B, Hertwig S 2012 The pYV virulence plasmids of *Yersinia pseudotuberculosis* and *Y. pestis* contain a conserved DNA region responsible for the mobilization by the self-transmissible plasmid pYE854. Environ Microbiol Rep 4:433–438. doi:10.1111/j.1758-2229.2012.00353.x. 23760829

[11] Hammerl JA, Klein I, Lanka E, Appel B, Hertwig S 2008 Genetic and functional properties of the self-transmissible *Yersinia enterocolitica* plasmid pYE854, which mobilizes the virulence plasmid pYV. J Bacteriol 190:991–1010. doi:10.1128/JB.01467-07. 18055592 PMC2223581

[12] Hertwig S, Klein I, Schmidt V, Beck S, Hammerl JA, Appel B 2003 Sequence analysis of the genome of the temperate *Yersinia enterocolitica* phage PY54. J Mol Biol 331:605–622. doi:10.1016/S0022-2836(03)00763-0. 12899832

